# A multi-source global-local model for epidemic management

**DOI:** 10.1371/journal.pone.0261650

**Published:** 2022-01-12

**Authors:** José Ulises Márquez Urbina, Graciela González Farías, L. Leticia Ramírez Ramírez, D. Iván Rodríguez González

**Affiliations:** 1 Unidad Monterrey, CIMAT, Monterrey, N.L., México; 2 Consejo Nacional de Ciencia y Tecnología, México City, CDMX, México; 3 Probability and Statistics, CIMAT, Guanajuato, Gto., México; 4 Department of Technological Services, CIMAT, Guanajuato, Gto., México; Politecnico di Torino, ITALY

## Abstract

The Effective Reproduction Number *R*_*t*_ provides essential information for the management of an epidemic/pandemic. Projecting *R*_*t*_ into the future could further assist in the management process. This article proposes a methodology based on exposure scenarios to perform such a procedure. The method utilizes a compartmental model and its adequate parametrization; a way to determine suitable parameters for this model in México’s case is detailed. In conjunction with the compartmental model, the projection of *R*_*t*_ permits estimating unobserved variables, such as the size of the asymptomatic population, and projecting into the future other relevant variables, like the active hospitalizations, using scenarios. The uses of the proposed methodologies are exemplified by analyzing the pandemic in a Mexican state; the main quantities derived from the compartmental model, such as the active and total cases, are included in the analysis. This article also presents a national summary based on the methodologies to illustrate how these procedures could be further exploited. The supporting information includes an application of the proposed methods to a metropolitan area to show that it also works well at other demographic disaggregation levels. The procedures developed in this article shed light on how to develop an effective surveillance system when information is incomplete and can be applied in cases other than México’s.

## Introduction

Simple compartmentalized epidemiological models have proven their usefulness for different infectious agents. Nevertheless, to model an infectious process at a regional or global level, it is important to consider the structure of subpopulations and their heterogeneous relations because, frequently, they are induced by the spatial structure and the associated mobility [1–4]. Following this approach, reaction-diffusion models on metapopulation networks have played an essential role in journals with a physics focus [5–7]. Even though several theoretical results can be derived, the parameter selection or statistical inference in these models has been done with numerically intensive methods like Markov Chain Monte Carlo (MCMC), Approximate Bayesian Computation (ABC), or Particle Filtering, that involve Monte Carlo simulations with algorithms such as Gillespie or Agent-Based [8, 9]. This characteristic prohibits their use for regions where the total population size is of the order of hundreds of millions of inhabitants.

This article considers a SEIRD compartmental epidemiological model that describes the most important transmission characteristics of COVID-19. It is assumed that the cities, or states, go through an epidemiological evolution described by the compartmental model and that such evolution is related to the inhabitants’ mobility between these locations. The mobility between subpopulations is integrated into the model through the evolution of the effective reproduction number *R*_*t*_ of each subpopulation and exposure scenarios. This number not only captures the intrinsic dynamics of the pandemics in a subpopulation and the impact of the interaction with other subpopulations, but it also captures the dynamics related to autonomous controls and the instruments that local and national governments implement, such as the social distancing of the inhabitants [10]. An essential aspect of the present work is that it employs exposure scenarios; such scenarios intend to reflect plausible conditions of the evolution of the pandemic.

The emergence of SARS-CoV-2 has motivated the publication of a plethora of epidemic models and methods to target specific questions based on particular local circumstances and available data. Many of these works highlight the importance of questions beyond the prediction of the number of cases and/or propose new models/methods to introduce relevant variables that characterize this virus, its evolution in the individuals, local population characteristics or local interventions implemented by governments. For example, in [11] the authors address the non-pharmaceutical intervention (NPI) known as test-trace-and-isolate (TTI), to study its effectiveness and the existence of a stable equilibrium at low case numbers. There are other works that propose epidemiological models used in conjunction of the analysis of scenarios. For example, [12] proposed a change point estimation for the transmission rate *β* of a SIR model, with a predefined number of change points. The scenario specification is relaxed using Bayesian MCMC and introducing prior distributions for the transmission rate associated with a percentage of decrease after each change point. The change point analysis of *R*_*t*_ (or *β*(*t*)) can reflect the encouraged social distancing and mobility restrictions; however as new controls are implemented, the marginal effects can be confounded in the resulting *R*_*t*_ (*β*(*t*)). Other works extend compartmental models to heterogeneous populations introducing compartments and parameters, for each subpopulation, that can describe the within and between contacts. These types of models, also known as patch models, have been studied in works such as [2, 13, 14]. Their findings shed light on the heterogeneity/mobility impact on the resulting *R*_0_, *R*_*t*_, but due to the large number of parameters usually involved, even for moderate numbers of subpopulations, performing statistical inference is prohibitive.

The main goal of this work is to introduce a tool, based on exposure scenarios, for regional epidemic management under *incomplete data*. The tool consists of a SEIRD compartmental model with a parametrization based on age groups and data from different publicly available sources. In this context, incomplete data means a situation where not all the data necessary to satisfactorily estimate the SEIRD model are available. The proposed parametrization allows that the parameters in the SEIRD model can be elicited from other sources. The data employed in such a parametrization comprises mobility, population density, the prevalence of comorbidities in a region, among others.

The reparametrized SEIRD model in conjunction with its confidence bands is called the epidemiological calculator (EC). The EC provides approximations of the evolution of several essential features that could assist the authorities in managing an epidemic. These features include, but are not restricted to, active cases, the total population infected at the end of the pandemic, and the number of deaths. The EC also allows a better evaluation of the time-dependent effective reproduction number and computing better approximations of crossings of such features like active and hospitalized cases by group age. All these curves contain 95% confidence bands, based on functional data analysis, to measure their level of uncertainty.

**Fig 1:**
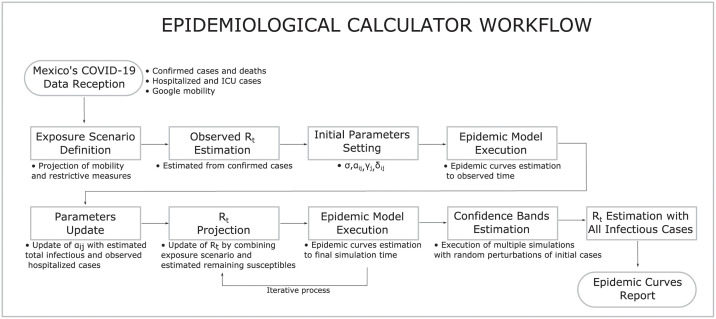
EC workflow. The figure presents the workflow for the EC.

A complete list of the input and output data for the SEIRD model and its parametrization is presented in Tables 1–7. Besides, the workflow of the EC appears in Fig 1. The process starts with the reception and cleaning of México’s COVID-19 data. Such data includes, at the municipal level, information of daily number of confirmed cases and deaths, the occupancy of regular and ICU beds in hospitals, and Google’s mobility information. The next step is the definition of exposure scenarios taking into account the most recent events and conditions for the pandemic for each region under consideration. After this, *R*_*t*_ is estimated from the pandemic’s beginning to the current date using confirmed cases’ data. The next action in the workflow is setting the initial parameters for the SEIRD model using proper reparametrization in terms of observable available quantities. Then, the SEIRD model is run to obtain an initial approximation of the epidemic curves. Some parameters are updated using the information of the first run of the epidemic model (EM). Then, *R*_*t*_ is projected into the future through a proper weighting of the exposure scenario and the remaining susceptible population, which produces an *R*_*t*_ curve for all the pandemics. This *R*_*t*_ curve is employed for a new run of the EM, which generates a new curve for the susceptible population. This new curve for the susceptible population is used for a new projection of *R*_*t*_ through the weighting defined before. This process of running the EM and projecting *R*_*t*_ is repeated until *R*_*t*_ converges, which means future projections remain unchanged. With the converging *R*_*t*_, a final run of the EM is obtained. Using this last *R*_*t*_ and the EM, epidemic curves are simulated inducing random perturbation on the initial cases; these curves are employed to determine 95% confidence bands using functional data analysis. Besides, the epidemic curves of the last run of the EC are used to recalculate a final *R*_*t*_ employing [15] and generate all the possible reported quantities.

**Table 1:** SEIRD model parameters based on Nuevo León.

Parameter	Epidemiologic Meaning	Value	Calculation
*β*	Contagious rate	Time-varying	Estimated from *R*_*t*_
*σ*	Latency period distribution rate	0.36 (mean 5.56 days)	Left constant (explored and adjusted from data and literature)
*α*_*i*1_	Probability of being asymptomatic for age group *i*	Time-varying	Fitted from observed data (see Eq 4)
*α*_*i*2_	Probability of having mild symptoms for age group *i*	Time-varying	Fitted from observed data (see Eq 4)
*α*_*i*3_	Probability of being hospitalized in normal bed for age group *i*	Time-varying	Fitted from observed data (IRAG [36])
*α*_*i*4_	Probability of being hospitalized in ICU for age group *i*	Time-varying	Fitted from observed data (IRAG [36])
*γ*_1_	Infectious period distribution rate for asymptomatic cases	0.1666 (mean 12 days)	Left constant (explored and adjusted from data and literature)
*γ*_2_	Infectious period distribution rate for mild symptoms cases	0.1666 (mean 12 days)	Left constant (explored and adjusted from data and literature)
*γ*_3_	Infectious period distribution rate for hospitalized cases in normal bed	0.1405 (mean 14.24 days)	Left constant (explored and adjusted from data and literature)
*γ*_4_	Infectious period distribution rate for hospitalized cases in ICU	0.1405 (mean 14.24 days)	Left constant (explored and adjusted from data and literature)
*δ*_*i*1_	Probability of death for asymptomatic cases for age group *i*	0 (no deaths for asymptomatic)	Left constant
*δ*_*i*2_	Probability of death for mild symptoms cases for age group *i*	Time-varying	Fitted from observed data (COVID-19 data [26], excess mortality [33])
*δ*_*i*3_	Probability of death for hospitalized cases in normal bed for age group *i*	Time-varying	Fitted from observed data (COVID-19 data [26])
*δ*_*i*4_	Probability of death for hospitalized cases in ICU for age group *i*	Time-varying	Fitted from observed data (COVID-19 data [26])
*N*	Total population size	5, 610, 153	Left constant [31]
*N*_*i*_	Population of age group *i*	*N*_1_ = 915, 056 *i* *N*_2_ = 4, 080, 198 *N*_3_ = 614, 899	Left constant [31]

**Table 2:** SEIRD model variables.

Variable	Epidemiologic Meaning
*S*_*i*_	Total remaining susceptible in age group *i*
*E*_*i*_	Exposed individuals in age group *i*
$I_{i1},I_{i1}^{\prime }$	Active infectious cases that remain asymptomatic in age group *i*
$I_{i2},I_{i2}^{\prime }$	Active infectious cases that have mild symptoms in age group *i*
$I_{i3},I_{i3}^{\prime }$	Active infectious cases hospitalized in normal bed in age group *i*
$I_{i4},I_{i4}^{\prime }$	Active infectious cases hospitalized in ICU bed in age group *i*
*Y*_*i*1_	Total accumulated cases that remained asymptomatic in age group *i*
*Y*_*i*2_	Total accumulated cases that had mild symptoms in age group *i*
*Y*_*i*3_	Total accumulated cases hospitalized in normal bed in age group *i*
*Y*_*i*4_	Total accumulated cases hospitalized in ICU bed in age group *i*
*R*_*i*_	Total recovered cases in age group *i*
*D*_*i*_	Total deceased cases in age group *i*

**Table 3:** EC parameters based in Nuevo León.

Parameter	Epidemiologic Meaning	Value	Calculation
*K*	Adjustment for excess mortality	Depends on population density	Explored and assumed (excess mortality [33, 35], population density [31, 44])
*Morb*_*i*_	Estimated morbidity for diseases that increase the risk of developing severe COVID-19	*Morb*_1_ = 0.0092 *Morb*_2_ = 0.0190 *Morb*_3_ = 0.0341	Inferred from observed data (morbidity [32], mortality [34])
*p*_*i*3_	Proportion of normal beds from observed hospitalized cases in age group *i*	0.778	Calculated from observed data (IRAG [36])
*p*_*i*4_	Proportion of ICU beds from observed hospitalized cases in age group *i*	0.222	Calculated from observed data (IRAG [36])
*p*_*wi*_	Proportion of not hospitalized cases that remain asymptomatic	*p*_*w*1_ = 0.7 *p*_*w*2_ = 0.6 *p*_*w*1_ = 0.5	Derived from literature
*N*_*pert*_	Number of perturbations to generate confidence bands	100	Left constant

**Table 4:** EC inputs.

Parameter	Epidemiologic Meaning	Value	Calculation
*R*_*t*_	Effective reproduction number at time *t*	Time-varying	Estimated from observed data
*Exp*_*t*_	Level of exposure to COVID-19 at time *t* (defines exposure scenario)	Time-varying	Explored (Proposed)
*M*_*t*_	Percentage decrease in mobility *t*	Time-varying	Shared by the research center INFOTEC
*C*_*i*_	Observed confirmed cases for age group *i*	Time-varying	Retrieved from México’s official COVID-19 platform
*H*_*i*_	Observed confirmed cases in hospital for age group *i*	Time-varying	Retrieved from México’s official IRAG system
*ICU*_*i*_	Observed confirmed cases in ICU for age group *i*	Time-varying	Retrieved from México’s official IRAG system
*Def*_*i*_	Observed deceased cases for age group *i*	Time-varying	Retrieved from México’s official COVID-19 platform

**Table 5:** EC outputs.

Variable	Epidemiologic Meaning
*I*	Active infectious cases
*Y*	Total accumulated cases
*Is*_1_	Active infectious cases that remain asymptomatic
*Is*_2_	Active infectious cases that have mild symptoms
*Is*_3_	Active infectious cases hospitalized in normal bed
*Is*_4_	Active infectious cases hospitalized in ICU bed
*Ia*_*i*_	Active infectious cases in age group *i*
*H*	Active hospitalized cases in normal bed
*ICU*	Active hospitalized cases in ICU
*Ht*_*i*_	Active hospitalized cases (both in normal bed and ICU) in age group *i*
*D*	Total deceased cases
*D*_*i*_	Total deceased cases in age group *i*
*S*	Remaining susceptible
*R*	Total recovered cases
*RI*_*t*_	Effective reproductive number with all infected cases

**Table 6:** *R*_*t*_ parameters.

Parameter	Epidemiologic Meaning	Value
*σ*	Standard deviation for the proposed normal distribution for the a priori probability	Fitted
*γ*	Serial interval for the proposed Poisson distribution for likelihood	1/12 Left constant
*R*_0_	Basic reproduction number	Left constant
*d*	Number of omitted days at the end of the new daily cases time series	12 days Left constant
*s*	Window size of moving average for smoothing the new daily cases time series	7 days Left constant

**Table 7:** *R*_*t*_ variables.

Variable	Epidemiologic Meaning	Value
*k*_*t*_	Times series of new daily confirmed COVID-19 cases by first symptoms date	Observed
$k_{t}^{s}$	Smoothed times series of new daily confirmed COVID-19 cases by first symptoms date	Observed
$k_{t}^{a}$	Times series of new daily confirmed COVID-19 cases by first symptoms date adjusted for symptoms to confirmation delay	Observed
*Delay*	Distribution of days passed between first symptoms and COVID-19 confirmation	Inferred from observed data
*R*_*t*_	Estimated effective reproduction number	Inferred from observed data

In México, as in most countries, there were delays in data availability regarding the pandemic’s evolution that hindered the decision-making process. On the other hand, some vital data required to get a realistic picture of the pandemic was unavailable. For example, there was no screening test data to estimate the actual size of the pandemic. These situations created an incorrect perception of some fundamental aspects of the pandemic; for instance, the Mexican COVID-19 mortality rate was inflated due to the lack of this information. The EC aims to present a better, more realistic picture of an epidemic/pandemic in scenarios with incomplete data as those described above. For example, it provides approximations of the size of the infected population, including undetected cases, and for the level of stress for the local and national healthcare systems. Thus, the information produced by the EC supports the decision-making process helping to detect regions requiring more resources and more stringent social distancing measures.

The epidemiological model proposed in this article assumes that those who recover will enjoy a period during which they will be immune. Nevertheless, COVID-19 reinfection cases have been reported [16] and it could require a modification of the proposed model [17]. The vaccination campaign implemented in several countries will also affect the pandemic’s dynamics, and it may be necessary to incorporate it into the model. The models and the methodologies proposed in this article can be generalized to include the effect of reinfections and vaccination. The updating of the newly susceptible and the schedule of the vaccine’s effects will be accomplished in a future publication. The data to update the model are not yet available in México (February 5th, 2021), and it is not clear when they will be.

The section “Models and Methods” presents a SEIRD model parametrized with a specific reparametrization. To exemplify the proposed methodologies, the section “Results and Discussion” presents results obtained from the EC in two examples. In the supplemental material, the proposed EC is applied to the Metropolitan Area of México City. It is important to remark that despite the procedures developed in this article are based on México’s case, the ideas and procedures can be adapted to other frameworks with incomplete data.

## Methods

### Epidemic model

The base epidemiological model consists in a compartmental SEIRD model with which each of the subpopulations of interest (states and metropolitan areas) are modeled. This model is numerically solved given the initial conditions and the values of the parameters.

The local model is based on a SEIRD compartmental model, to which we introduce some synthetic compartments *I*′*s*. This allows the consideration of the distribution of time infectivity with an Erlang distribution. This distribution can capture in a more realistic form these epidemiological periods and, even though the model here presented considers an Erlang distribution with parameter of *k* equals 2, it can be modified easily to consider an arbitrary value of *k*.

**Fig 2:**
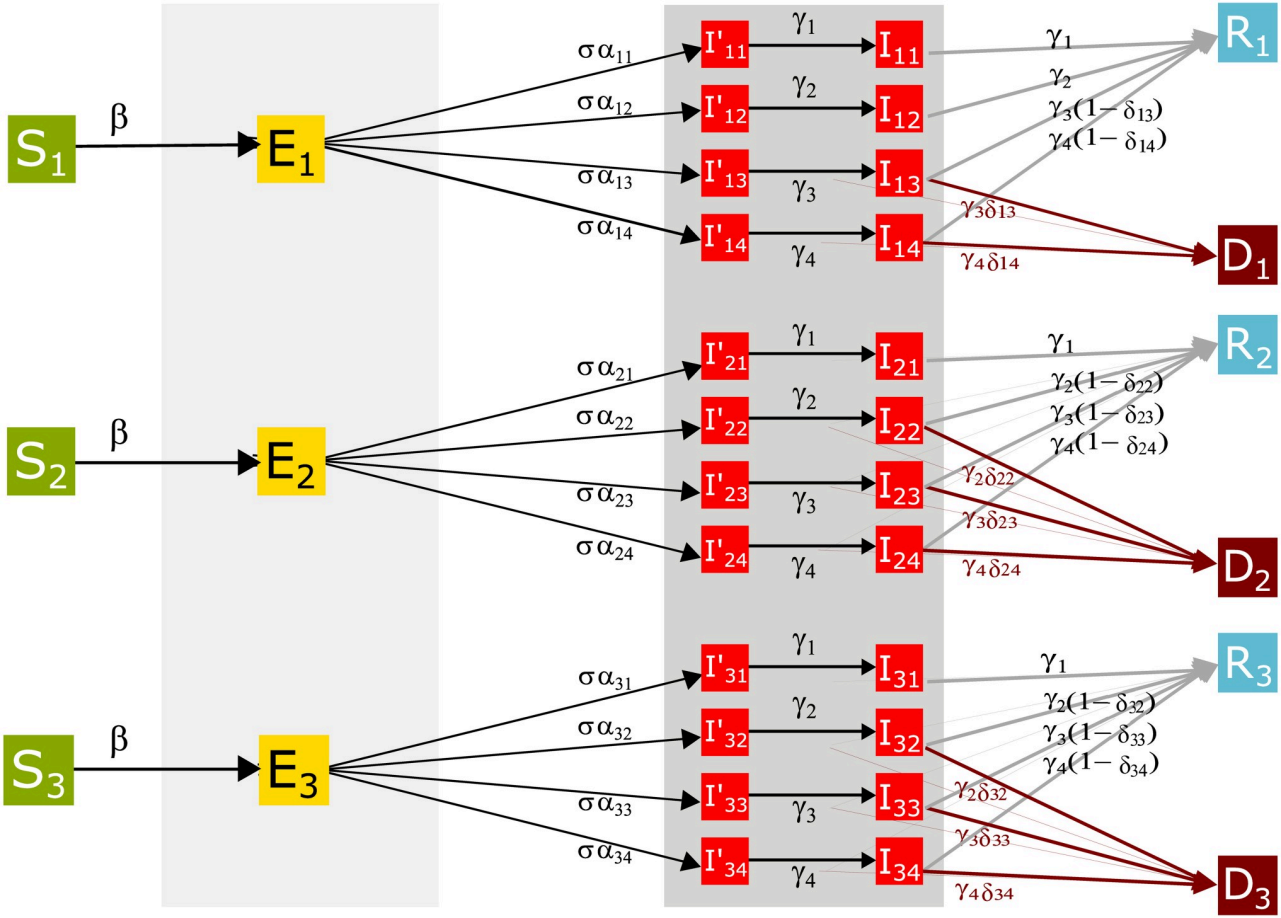
Compartmental SEIRD model for México. The diagram presents the compartmental model to describe COVID-19’s dynamic in México divided by subpopulations.

The local model is applied to each of the *m* subpopulations, each of them composed by three subsubpopulations defined by age groups. For each region, the sociodemographic information is considered to establish the composition of the age groups: 0–9, 10–60 and 60–+ years old. Fig 2 represents the compartmental model for a region; the model is formally expressed with the equation at (1) which corresponds to the dynamic for the *i*-th age group (*i* = 1, 2, 3).

$$\begin{array}{c} \begin{array}{c} \begin{array}{cc} {\dot{S}}_{i} & =-\beta \left(t\right)S_{i}I^{⋆}/N\\ {\dot{E}}_{i} & =\beta \left(t\right)S_{i}I^{⋆}/N-\sigma_{i}E_{i}\\ {\dot{I}}_{i1}^{\prime } & =\sigma_{i}\alpha_{i1}E_{i}-\gamma_{1}I_{i1}^{\prime }\\ {\dot{I}}_{i2}^{\prime } & =\sigma_{i}\alpha_{i2}E_{i}-\gamma_{2}I_{i2}^{\prime }\\ {\dot{I}}_{i3}^{\prime } & =\sigma_{i}\alpha_{i3}E_{i}-\gamma_{3}I_{i3}^{\prime }\\ {\dot{I}}_{i4}^{\prime } & =\sigma_{i}\alpha_{i4}E_{i}-\gamma_{4}I_{i4}^{\prime }\\ {\dot{I}}_{i1} & =\gamma_{1}I_{i1}^{\prime }-\gamma_{1}I_{i1}\\ {\dot{I}}_{i2} & =\gamma_{2}I_{i2}^{\prime }-\gamma_{2}I_{i2}\\ {\dot{I}}_{i3} & =\gamma_{3}I_{i3}^{\prime }-\gamma_{3}I_{i3}\\ {\dot{I}}_{i4} & =\gamma_{4}I_{i4}^{\prime }-\gamma_{4}I_{i4}\\ {\dot{D}}_{i} & =\gamma_{2}\delta_{i2}I_{i2}+\gamma_{3}\delta_{i3}I_{i3}+\gamma_{4}\delta_{i4}I_{i4}\\ {\dot{R}}_{i} & =\gamma_{1}I_{i1}+\gamma_{2}\left(1-\delta_{i2}\right)I_{i2}+\gamma_{3}\left(1-\delta_{i3}\right)I_{i3}+\gamma_{4}\left(1-\delta_{i4}\right)I_{i4}\\ & \text{where}\;\delta_{12}=1\;\text{and}\;0<\delta_{i2}<1\;\text{for}\;i=2,3. \end{array} \end{array}\} \end{array}$$ (1)

It is assumed that within a population of size *N*, the infectious contacts for the group of ages *i* result from the interaction between those who are susceptible in *i* and all those infected in the population across age groups, identified as *I*^⋆^. Then *I*^⋆^ is the total number of infectious individuals who are not in the hospital. This total is across all the severities and age groups, so we assume that the contact pattern is homogeneous among these groups. At the end of the latent (exposure) period, the individuals become infectious and this status is divided into four possible illness scenarios that are defined by the severity of the disease. It is assumed that the individuals can be asymptomatic infected (*I*_1_), experience mild symptoms (*I*_2_), develop more severe symptoms that will require hospitalization in regular bed (*I*_3_), or will require attention in an intensive care unit (*I*_4_). It is assumed that the distribution of the exposed individuals that will develop the different levels of illness can vary by age group [18]. Thus *α*_*ij*_ > 0, *i* = 1, 2, 3, *j* = 1, 2, 3, 4 where ∑_*j*_ *α*_*ij*_ = 1 for *i* = 1, 2, 3.

Since the severity of the illness may correspond to different infectious periods [19], we considered four different rates *γ*_1_, *γ*_2_, *γ*_3_ and *γ*_4_ for the individuals to be able to complete this period.

Finally, at the end of the infectious period we assume that an individual can recover (*R*) or they die (*D*). Following the results reported in the literature, like [20], at the beginning we considered that only the individuals who have experienced important or severe symptoms could die. However, given the saturation of hospitals and the consequent deaths of infected individuals at home, the model was modified to include the possibility that those returning home could expire. Thus, we assume that the individuals that require hospital attention or an intensive care unit die with probabilities that vary according to the severity of illness and age group *δ*_*ij*_ > 0, *i* = 1, 2, 3, *j* = 3, 4; and that the people in the age groups *i* = 2, 3 with mild symptoms also can die, with probabilities *δ*_*i*2_ > 0.

The model proposed seeks to capture the variations of the most important parameters by age groups while keeping the model parsimonious. For instance, as initially mentioned, contagions are considered as the effect of a common rate in a population that is mixing without considering preferences by age groups.

Given the initial conditions and the values of the parameters, the model is solved using the Runge-Kutta method of order 4 implemented in R [21]. The numeric solution enables incorporating non-constant values for *β*, *σ* and *γ*_*j*_, *j* = 1, 2, 3, 4, which can be introduced as functions evaluated at discrete times *t*_0_, *t*_1_, …. Such functions may correspond to constant functions at certain time intervals, or the values can be linearly interpolated between values at consecutive time points to obtain a continuous function. This is detailed later. Considering parameters that vary with time allows contemplating the effects of intervention measures and/or the effect on the evolution of other related subpopulations. This important element of the global model will be described later.

For a region *r*, the SEIRD model with the proper parametrization and confidence bands is called the epidemiological calculator (EC) for *r*. As pointed out before, the EC provides approximations of the evolution of several essential features that could assist the authorities in managing an epidemic. For regions divided into disjoint subregions, each with a local EC, the conjunction of all the local ECs is called the global EC. For example, the ECs for the states/departments in a country combine into a (global) national EC. Adding the local ECs makes it possible to obtain global approximations of the same quantities that the local ECs provide. In the following discussions, when considering the EC, it is assumed that it is a local EC unless stated differently.

## México case: An application

We present a way to adjust the parameters in the SEIRD model using the official information available for México. The methodology results in a reparametrization of the SEIRD model in terms of observable quantities. This reparametrized SEIRD model, in conjunction with the confidence bands, is what defines the EC.

The calculator may have multiple applications. In this article, it is used to obtain local (state) and global (national) projections of some of the variables that help manage an epidemic. Such a projection is based on scenarios for the local evolutions (states). For example, for a given exposure scenario, the EC permits estimating the group of asymptomatic, light symptoms, or hospitalized cases for each age group; also, it allows to count their consequences in deaths, recovered, and the remaining susceptible population. It is important to mention that the EC can be initialized even at the early pandemic stages because it permits updating its parameters as the pandemic evolves. In the supplemental material, the EC is applied to the Valle de México metropolitan area to show that it also works at other demographic disaggregation levels.

To initialize the calculator, notice that the value of the transmission parameter *β* ≡ *β*(*t*) can be established as a function of *R*_*t*_ [22, 23]. Considering that *R*_*t*_ moves faster to zero than the number of susceptible available, the representation chosen in the present work is

$$\begin{array}{c} \beta \left(t\right)=C\cdot R_{t}\times \frac{N}{S(t)}, \end{array}$$ (2)

where *N* is the size of the susceptible population and *C* is a positive constant. After multiple analysis, it was found that C=1/E provides the best fit, where E represents the expectation of the infectious period.

**Fig 3:**
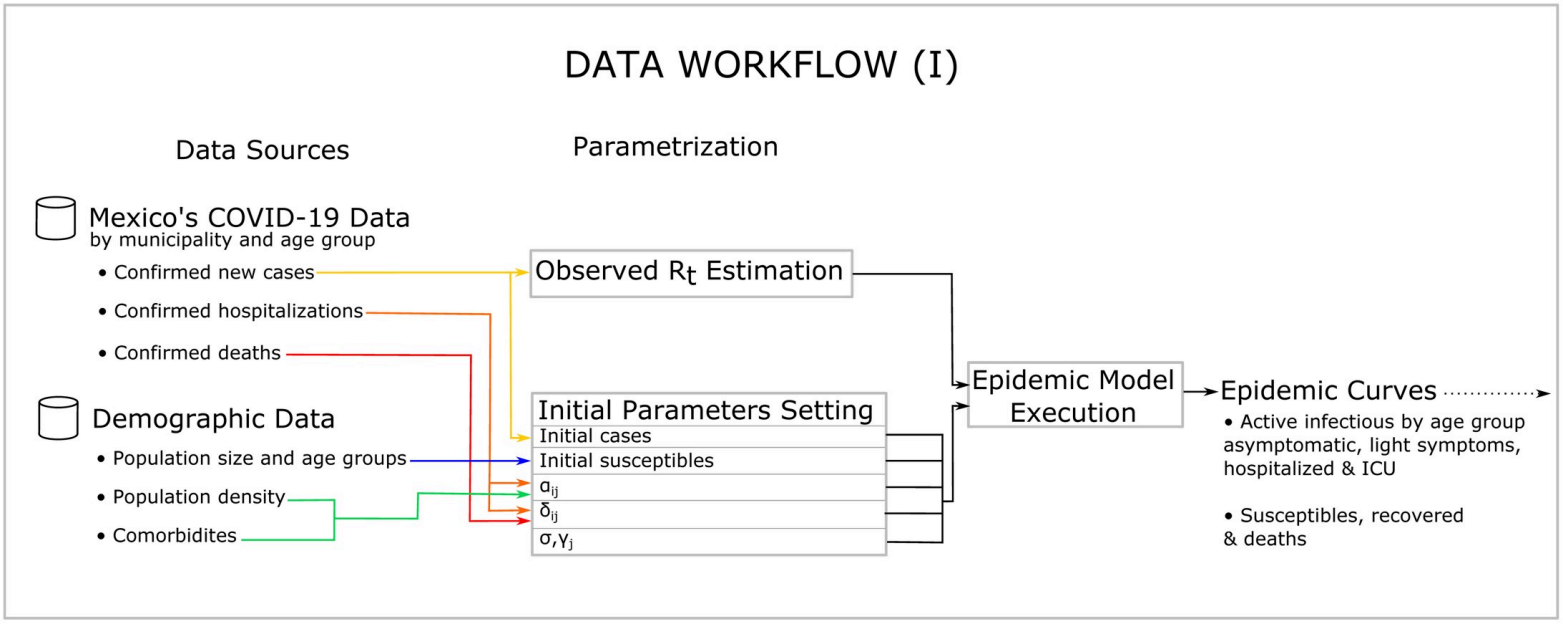
Data workflow 1.

**Fig 4:**
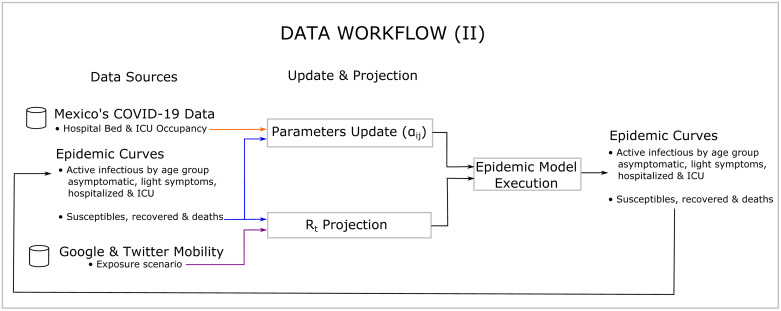
Data workflow 2.

The EC also requires initial information for each stratum about the population density, the health condition of the population by age group, and excess mortality to initialize its parameters. A complete list of the required information is listed in Figs 3 and 4. The population’s health condition is measured by the prevalence of the main comorbidities associated with COVID-19—all of these in accordance with reports from different scientific sources. The EC also requires the specification of an exposure scenario which is used to project *R*_*t*_ and the epidemic curves into the future. The motivation to include in the EC all the information listed in Figs 3 and 4 comes from reports in the literature suggesting they influence the evolution of the pandemic.

It is important to emphasize that, in strictly statistical terms, the EC’s parameters are not estimated. However, proposals about calculating them from external sources and the pandemic’s behavior in time are presented. In this context, we should take into consideration that, for México and for a long time, the only available information was the number of hospitalized cases. Fig 1 presents the complete workflow associated with the EC. Later in the section we describe more detail about the figure. In the following, we describe how mobility information is employed to project *R*_*t*_ into the future and how all the parameters in the SEIRD model are adjusted.

### Effective reproduction number estimation and projection

A fundamental input to obtain the EC is the contagion rate given by *R*_*t*_ for the whole duration of the pandemic; that is, it is necessary to determine the observed *R*_*t*_ and its projection to the future. In the following, is described the process to compute the observed effective reproduction number *R*_*t*_ and how it is projected into the future based on exposure scenarios.

#### Observed *R*_*t*_

**Fig 5:**
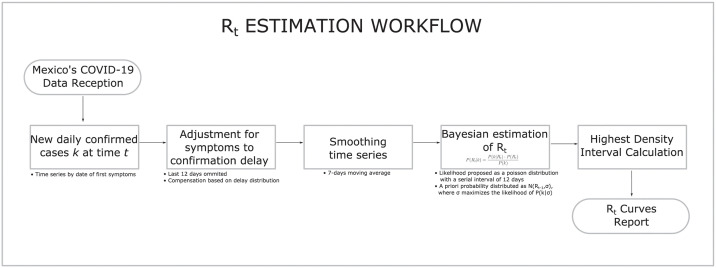
Observed *R*_*t*_ workflow.

The effective reproduction number *R*_*t*_ is a temporal parameter that is calculated using the Bayesian procedure proposed in [15], based on the elicitation of the parameter *σ*; the method presented [15] is a dynamic modification of the method developed in [24]. Fig 5 presents the complete workflow for *R*_*t*_ associated with the EC. The proposal in [15] is based on the number of new symptomatic COVID-19’s cases ${\left\{k_{t}\right\}}_{t\in T}$, T=0,1,…,K, where *K* is the maximum number of days observed since the beginning of the outbreak.

The procedure also considers that in a particular day *t*, *k*_*t*_ depends on the effective reproduction number *R*_*t*_ of that day. To model such dependence, it is assumed that *k*_*t*_|*R*_*t*_ ∼ *Poisson*(λ_*t*_) with λ_*t*_ = *k*_*t*−1_ exp (*νR*_*t*−1_), where *ν* is the reciprocal of the serial interval. For México we consider a serial interval of 12 days, i.e. *ν* = 1/12, in contrast with the 7 days assumed by the original publication. The serial interval varies from country to country, in accordance with public policies, as it has been reported in [25].

The method in [15] also assumes that the effective reproduction number *R*_*t*_ at day *t* depends on *R*_*t*−1_ through *R*_*t*_|*R*_*t*−1_ ∼ *Normal*(*R*_*t*−1_, *σ*), where it is used *Normal*(*μ*, *ρ*) to denote a normal density with mean *μ* and variance *ρ*^2^. For this continuous-time Hidden Markov Model, the value of *σ* is selected to maximize the likelihood of *P*(*k*_0_) ⋯ *P*(*k*_*K*_) for the interval of *σ* proposed. In our case, we observed that it was better to maximize the variance of each stratum and not the maximum variance of all of the strata. The estimation process is updated as new information becomes available.

Thus, knowing *R*_0_ and the number of new daily cases ${\left\{k_{t}\right\}}_{t\in T}$ and using the Bayes Theorem and the distributions of *k*_*t*_|*R*_*t*_ and *R*_*t*_|*R*_*t*−1_, it is possible to calculate the posterior distribution of *R*_*t*_|*k*_*t*_. It must be noted that in the reference [15] is used the same value of *R*_0_ (=3) for all of the states of the USA; however, in the case of México, the value of *R*_0_ was recalculated for each state because of the extreme heterogeneity among them.

The new daily cases by the date of the first symptoms are provided by the platform of open data of the Mexican Government [26]. To compensate for the days that pass between the beginning of the symptoms and the date of confirmation of COVID-19, the last twelve days are eliminated to adjust the calculator and *R*_*t*_. The data of daily new cases is smoothed with a weighted moving average.

#### Projection of *R*_*t*_: *R*_*t*_, mobility, and exposure scenarios

This subsection discusses how to project *R*_*t*_ into the future based on exposure scenarios. The exposure scenarios are constructed from mobility information and the public policies regarding the limitation of social activities. Discussions highlighting the relation between mobility and *R*_*t*_ and the limitations of only consider mobility for projecting *R*_*t*_ are included.

From the beginning of the pandemic, we searched for reliable sources of mobility information. Given the public policies that restricted mobility, the purpose was to evaluate the efficacy of such measures. The data obtained was provided by Google and Twitter -for more details on this mobility data, see [27, 28]. We found that the effective reproduction number *R*_*t*_ is correlated with mobility, as reported by [29]. However, as time passes, this correlation decreases [30]. This situation is caused by a decrement in the size of the susceptible population. Thus, an accurate description of *R*_*t*_ should include both mobility and the size of the susceptible population. This point will be critical for maintaining the EC updated for the different phases of this pandemic.

The effective reproduction number *R*_*t*_ can be predicted from the rate of mobility previously described using a linear regression with Autoregressive Moving Average (ARMA) errors. However, it is well known that these models can provide only short-term predictions. The supporting information includes an exercise where *R*_*t*_ is predicted using a regression model with ARMA errors. However, the exercise provided us with a good insight of how to construct a more reliable *R*_*t*_ measurements taking in account the number of susceptible people under study.

As discussed, it is not possible to perform a prediction for *R*_*t*_. In this work, instead of predicting such a quantity, it is proposed to project *R*_*t*_ into the future in terms of plausible exposure scenarios. These scenarios are constructed from the mobility information, the remaining susceptible population, and the announcements of openings proposed by the state and federal governments. For the construction of such scenarios, mobility was considered only to the point where it has been observed.

**Fig 6:**
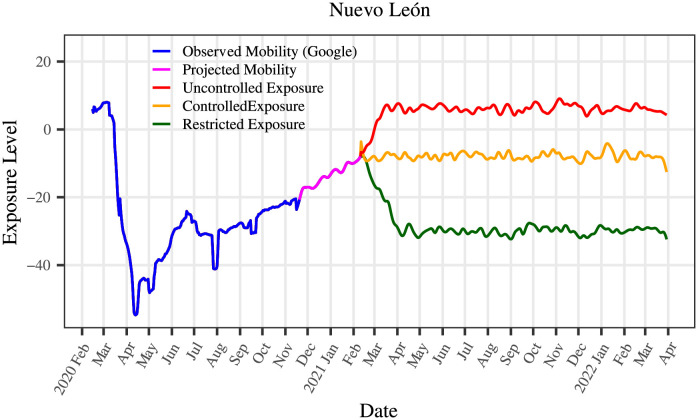
Exposure scenarios for the state of Nuevo León. The figure presents the observed mobility up to November 19th, 2020; from that point, it shows a projection of the mobility until the most recent date of this study (February 10th, 2021), followed by the three exposure scenarios.

In Fig 6, three exposure scenarios are presented: the first, in green, represents a scenario in which the population reduces its exposure to the virus through strong distancing measures and additional precautions such as hygiene measures and the use of face masks; the second, in orange, exhibits a scenario with measures of social distancing in those activities where it can be implemented; and the third, in red, presents a scenario in which no measures to limit the contagions are applied. These ideas are used to implement the effect of mobility, public policies, and susceptible populations interacting jointly. The construction of the scenarios was inspired by the mobility’s weekly variability before the pandemic and during the strictest confinement measurements. The first scenario is called the restrictive scenario, the second one the controlled scenario, and the third one is the uncontrolled scenario, respectively. The uncontrolled and the restricted scenarios are defined based on the dynamics before the pandemic and during the strictest confinement measurements, respectively; we used the average functional trend of the observed *R*_*t*_ for the controlled scenario. The results obtained in this work through the EC strongly depend on these scenarios. Thus, it is required to be constantly monitoring public policies in each of the population strata under study to be able to calculate them appropriately.

A projection of *R*_*t*_ can be computed through a weighted average of the exposure scenario and the size of the susceptible population. This procedure requires knowing the size of the susceptible population in time. Since the susceptible population is unknown, an iterative methodology to project *R*_*t*_ using the EC is discussed later. Fig 1 presents a general description of such a method.

In the following section is discussed a reparametrization of the SEIRD model in the EC. That is, a method to select the parameters *β*, *σ*, *α*_*ij*_, *γ*_*j*_ and *δ*_*ij*_, *i* = 1, 2, 3, *j* = 1, 2, 3, 4 from the available information for México’s case. To do that, the initial value of the parameters based on several information sources and international studies are described, and later, they are changed as functions in time, as described in the next section.

### Parameters for the epidemiological calculator

Fig 2 shows all the parameters that need to be adjusted in the SEIRD model in which the EC is based. In the following, the parameters’ adjustment using available epidemiological, demographic, mobility, and other types of information for México is presented. As already has been mentioned, some of these parameters are constants, and others are functions of time. All the necessary information is contained in the databases available in [26–28, 31–35]. The information contained in IRAG’s network is also used; a summarized version of the data in this network can be found in [36], although the whole database is not public. A complete list of the variables, parameters, inputs, outputs and the information employed in the adjustment appears listed in Tables 1–7. Besides, Fig 1 shows the complete workflow associated to the adjustment of the EC.

#### Parameter *β*

As it has been mentioned before, the EC is designed to read the input parameter *β* as a time function. Given *R*_*t*_ for the whole duration of the pandemic, *β* is adjusted using (2). The functional relation (2) between the effective reproduction number *R*_*t*_ and *β* is used to be able to describe the contagion dynamics. This transmission process depends, at time *t*, on the mobility, the effective reproduction number *R*_*t*_, and the size of the susceptible population under study. In addition, the relation (2) provides us with a stop rule for the input of new cases into the EC.

It is necessary to determine the observed *R*_*t*_ and its projection into the future to determine *β*. The previous section detailed how the observed *R*_*t*_ is calculated. An iterative process was designed for projecting *R*_*t*_ into the future. This algorithm requires the observed *R*_*t*_, the observed mobility, and an exposure scenario. For the observed mobility, the indices provided by Twitter and Google [27, 28], as we explained before, were considered. The exposure scenarios used as input are analogous to those described in Fig 6. The initial projection of *R*_*t*_ is calculated considering the observed mobility and a plausible exposure scenario through a linear regression where the predictor is the observed *R*_*t*_. This is the initial value in the iterative process, every time the data is updated.

This initial projection of *R*_*t*_ is used as an input in the EC. This run of the EC produces a curve of the susceptible population, which is used for a new projection of *R*_*t*_ through a new weighting as the one defined before, producing a new curve $R_{t}^{\prime }$ of *R*_*t*_. This curve $R_{t}^{\prime }$ is used as a new input in the EC, and the previous steps are repeated to determine a new curve for *R*_*t*_. This process is repeated iteratively until the curve of the susceptible population remains unchanged, generating a converging adjustment for the curve *R*_*t*_. With the infected cases provided by the last run of the EC, the final version of *R*_*t*_ is calculated outside the EC with the same method used for the observed *R*_*t*_ [15].

The EC needs to stipulate 21 parameters to run, of which only 5 are not time functions. The following paragraphs describe a way to determine such parameters.

#### Parameters *σ* and *γ*_*j*_

As it was specified before, a susceptible person enters the calculator while the value of *β*, that depends on *R*_*t*_, allows it. For an individual in any of the compartments *S*_*i*_, *i* = 1, 2, 3 in the SEIRD model, the times to enter and leave the calculator are ruled by the parameters *σ*, *γ*_*i*_, *i* = 1, …, 4. These 5 parameters are not time-dependent but depend on the conditions of the compartmental model of the severity of the disease. Their values are assigned in accordance with several epidemiological national registries from the Mexican National Council of Science and Technology (CONACYT) (e.g. [26]) and others Mexican institutions such as Secretaría de Salud, IMSS, INEGI, and CONAPO. For this task, the Mexican data was also compared with international sources that were issuing reports almost daily, such as [25, 37, 38], along with multiple reports in Science, Nature, The Lancet, The New England Journal of Medicine, medRxiv, and reports from CDC, Georgia; and notes from well-respected international newspapers such as The Economist, Financial Times, The New York Times, among others. The previous information was complemented by data from websites such as https://ourworldindata.org/ and https://www.euromomo.eu/.

The parameter *σ* controls the distribution of the latency period between the moment an individual is exposed to the virus and the moment it becomes infectious. The parameters *γ*_*j*_, *j* = 1, ‥, 4, have a similar role for the time between the infection and its end (recovery or death) and depend on the compartment *I*_*ij*_ but not on the age group *i*. Table 2 presents a description of the compartments *I*_*ij*_; besides, Fig 2 illustrates the interpretation of *σ* and *γ*_*j*_.

#### Parameters *δ*_*ij*_

The parameters *δ*_*ij*_, *i* = 1, 2, 3, *j* = 1, 2, 3, 4, control the proportion at which the infected people recover or die. As with *β*, the parameters *δ*_*ij*_, in general, depend on time. The parameter *δ*_*i*1_ is always 0, while, for each stratum, the parameters *δ*_*i*3_ and *δ*_*i*4_ are calculated from the proportion of people hospitalized in regular beds or intensive care units that die by age group. For these computations, we consider the reported deaths that occurred until one month before the considered date, and the change in time is by week; thus, these parameters become dependent on time. The calculation is based on open official data from the Mexican Government [26]. Therefore, the parameters *δ*_*i*3_ and *δ*_*i*4_ are calculated as

$$\begin{array}{cc} \delta_{i3} & =\frac{\text{total}\;\text{deceased}\;\text{hospitalized}\;\text{patients}\;\text{in}\;\text{regular}\;\text{bed}\;\text{from}\;\text{group}{}_{i}}{\text{total}\;\text{hospitalized}\;\text{patients}\;\text{in}\;\text{regular}\;\text{bed}\;\text{in}\;\text{group}{}_{i}},\\ \delta_{i4} & =\frac{\text{total}\;\text{deceased}\;\text{hospitalized}\;\text{patients}\;\text{in}\;\text{ICU}\;\text{from}\;\text{group}{}_{i}}{\text{total}\;\text{hospitalized}\;\text{patients}\;\text{in}\;\text{ICU}\;\text{in}\;\text{group}{}_{i}}. \end{array}$$

On the other hand, because deaths at home among those with light symptoms began to be reported, this possibility is added to the model. This is done, for each stratum, through the equation

$$\begin{array}{cc} \delta_{i2} & =c\cdot \frac{\text{total}\;\text{non-hospitalized}\;\text{deaths}\;\text{in}\;\text{group}{}_{i}}{\text{total}\;\text{deaths}\;\text{in}\;\text{group}{}_{i}}, \end{array}$$

where *c* is a weighting constant necessary to approximate the excess mortality national curve within the corresponding group. As before, we consider the reported deaths that occurred until one month before the end of the reported data and by week. The constant *c* is considered to avoid being below the observed deaths.

In some Mexican states, there were no reported deaths in the first age group during part of the pandemic. Therefore, *δ*_13_ and *δ*_14_ are set to a value extremely small. In summary, the fact that *δ*_13_, *δ*_14_, *δ*_22_ and *δ*_32_ are playing a role at this stage of the pandemic (February 10th, 2021) reflects that the groups in the compartments of those that stay at home or are 0–9 years old have begun to report deaths.

The parameters *δ*_*ij*_ could also be adjusted to reflect the mortality associated with COVID-19’s sequelae. In México, there is not enough information and evidence in this regard yet; therefore, such data are not considered yet.

#### Parameters *α*_*ij*_

For each age group *i*, *α*_*ij*_ provides the severity of the disease. Because the infected population that stayed at home with no symptoms or mild symptoms is not directly observable, we control all the parameters *α*_*ij*_ through *α*_*i*3_ and *α*_*i*4_. The parameters *α*_*i*3_ and *α*_*i*4_ correspond to cases in regular hospital beds and those hospitalized who already are at ICU or ultimately will require it, respectively. The *α*_*i*3_ and *α*_*i*4_ were calibrated with data observed in public hospitals and information from several epidemiological sources that are specified later, while keeping in mind the sum $\sum_{j=1}^{4}\alpha_{ij}=1$. See Fig 3.

To select the most adequate parameters *α*_*ij*_, the method requires the determination of initial values. For *α*_*i*3_ and *α*_*i*4_ and the age groups *i* = 1, 2, 3, these starting values are calculated as

$$\begin{array}{cc} \alpha_{i3} & =K\cdot p_{3}\cdot {\text{Morb}}_{i},\\ \alpha_{i4} & =K\cdot p_{4}\cdot {\text{Morb}}_{i}, \end{array}$$ (3)

where *K* is a positive constant; *p*_3_ and *p*_4_ correspond to the proportions of occupied regular beds and ICUs, respectively; and Morb_*i*_ represents the prevalence of comorbidities of the age group *i*.

The constant *K* introduces an increment of hospitalizations by COVID-19 with respect to the comorbidity of 2019; it intends to reflect the excess of mortality reported by the international literature [33, 35]. For an accurate depiction of reality, this constant *K* is computed for each municipality, considering the population’s density [31]. The proportions *p*_3_ and *p*_4_ are calculated by stratum and age group from data reported by public hospitals, which is provided through the network IRAG (last update 2021–02-10). This data is not public; it is provided by México’s Secretariat of Health and the Mexican Council of Science and Technology (a summarized version can be found in [36]). The comorbidities Morb_*i*_ are included to reflect a greater risk of death or severe complications with COVID-19. These comorbidity indices are calculated for each age group *i* and using data from 2019 [32].

On the other hand, the initial values of *α*_*i*1_ and *α*_*i*2_, for the age groups *i* = 1, 2, 3, are calculated as

$$\begin{array}{cc} \alpha_{i1} & =p_{wi}\cdot \left(1-\alpha_{i3}-\alpha_{i4}\right),\\ \alpha_{i2} & =\left(1-p_{wi}\right)\cdot \left(1-\alpha_{i3}-\alpha_{i4}\right), \end{array}$$ (4)

where *p*_*w*1_ = 0.7 for age group 1, *p*_*w*2_ = 0.6 for group 2 and *p*_*w*3_ = 0.5 for group 3, based on what has been observed in different countries that have been suffering the impact of the pandemic for several months. These proportions maybe changed in case there are reinfections in age groups in which it was not expected that reinfections would occur.

Notice that from the previous discussions, it follows that the parameters *α*_*ij*_ vary from location to location.

**Fig 7:**
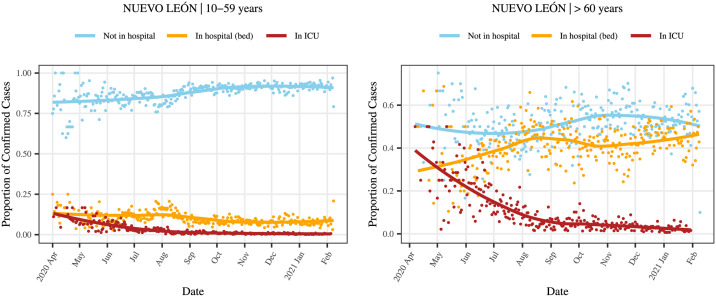
Proportion of the infected population by age group. The figure to the left presents the proportions of the infected population by the severity of the infection in the 10–59 age group. The figure on the right presents the same information for the 60 years and more age group. The red line represents the trend in persons that require intensive care, the yellow those that do not require a bed in intensive care and the light blue represents those with light symptoms that do not require hospitalization.

With the pandemic evolution and using the hospitalization registers available, *α*_*i*3_ and *α*_*i*4_ calculations allow the inclusion of information dependent on time and for the conversion of trends into functions. Specifically, the initial values of the parameters *α*_*ij*_ allow the obtention of a curve of the initial susceptible population. From such curve and the curve of hospital occupation given by the IRAG network until 2021–02-10, the values of *α*_*i*3_ and *α*_*i*4_ are updated using the proportion of susceptible who have required hospitalization (regular bed and ICU); that is, Eq (3) is substituted by such proportions that depend on time. Besides, Eq (4) continues be employed to compute *α*_*i*1_ and *α*_*i*2_, making them also dependent on time. The same procedure is used to project the values of *α*_*ij*_ until the end of the simulation for a particular scenario. Fig 7 illustrates the curves that serve as the base for these calculations. Note that those curves vary from stratum to stratum and by age group.

It is worth mentioning that the non-hospitalized population refers to the subpopulation of ambulatory patients, but it is important to consider that the output of non-hospitalized by the EC cannot be under that curve.

### Confidence bands

Given the initial conditions and the parameters’ values (or functions), the EC provides a curve that solves the epidemiological model (1). This solution does not provide a measure of the uncertainty for each one of the measurements derived from the EC. To consider a more realistic situation, the entrance of the susceptible population in the EC is perturbed, and confidence bands based on functional data analysis [39, 40] are constructed. These perturbations spread through all the epidemic curves and crossings of information.

**Fig 8:**
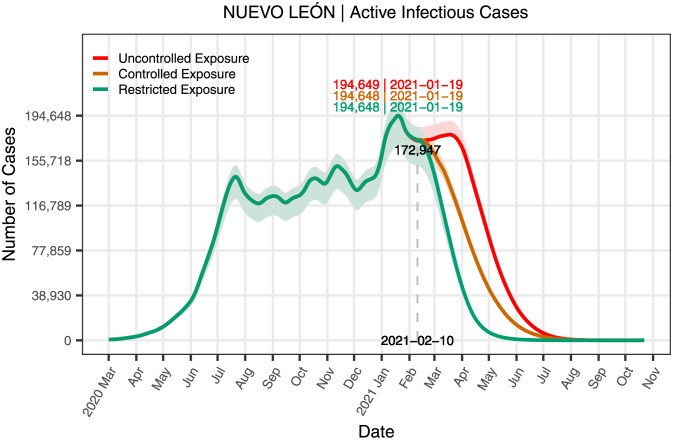
Active cases in the state of Nuevo León for three scenarios. The figure shows the curves of daily active infected cases resulting from the dynamics induced by each exposure scenarios.

The calculator provides approximations of multiple curves representing information for various aspects of the pandemic, e.g., the total number of infected individuals and deaths, and calculates confidence bands for whatever percentage is chosen. In Fig 8, it can be seen an example of 95% confidence bands. Specifically, to obtain these bands, an order induced by the notion of depth is defined. The resulting order is a centrality measure, and it can be thought of as an order statistic for curves. Given the definition of depth, the 95% of the deepest curves are considered in the construction of 95% confidence bands. The confidence region is defined as the band delimited by the maximum and the minimum of the deepest curves. In other words, the confidence region is the smallest band that includes 95% of the deepest curves.

Whatever the EC’s application may be, it must be emphasized that the initial parameters are calculated from external and complementary sources that inform key aspects of the pandemic dynamics in the population under study. Thus, confidence bands for the parameters of the EC are not needed.

## Results and discussion

In this section, the EC is applied to the federal states of México. Only the case of the state of Nuevo León is analyzed in detail. In addition, a national summary based on all Mexican states is presented. The state of Nuevo León is selected because it includes zones that vary from high to low population density and for having a metropolitan area with high international exchange, outstanding industry, banking, and commerce. Besides, Nuevo León has an important public and private hospital infrastructure, and the local authorities have implemented a reasonable monitoring system during the pandemic.

To show that the proposed EC also works at other strata and demographic disaggregation levels, in the supplemental material, it is presented the case of the Metropolitan Area of México City, known as Valle de México (Valley of México). This area was selected because of its national importance as the country’s capital and for being the most populated urban zone in México; its inhabitants represent 17% of the national population.

### Nuevo León

Under the scenarios illustrated in Fig 6, the following are some of the illustrative results produced by EC for the state of Nuevo León. It includes relevant information about the pandemic’s evolution that can be determined through the projected *R*_*t*_ curve. It is important to mention that the EC only considers and employs the information up to February 10th, 2021. From this date on, many significant changes occurred, like new variants, vaccination programs, and reinfections. The EC can take this information. However, there was no public information on these changes for México when this work was subject to the first revision.

When analyzing the susceptible population’s curve’s rate of change, it is possible to determine an endpoint for the pandemic for each of the scenarios. Specifically, it is said that the pandemic reaches an endpoint when the curve of the number of susceptible individuals remains almost constant during a prolonged time. Each exposure scenario leads to an endpoint different from the endpoint induced by the other two scenarios. One needs to be careful when speaking about the endpoint of the pandemic because many factors determine when a pandemic ends. For instance, if a new variant appears, the pandemic can regain strength, as we have seen recently.

**Fig 9:**
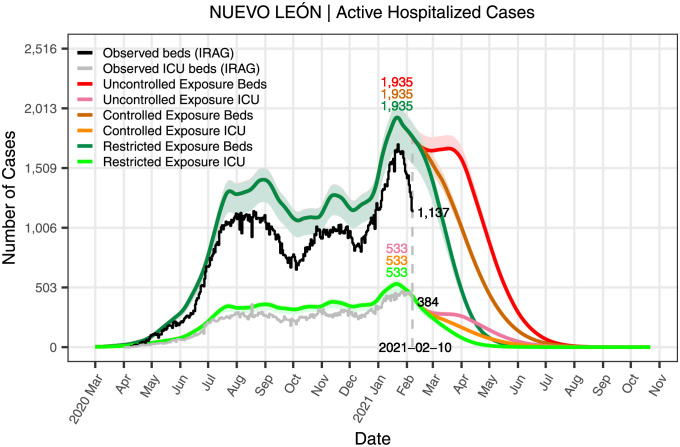
Active hospitalized cases in the state of Nuevo León for the three scenarios. The figure shows curves for the active hospitalized cases for each type of hospitalization (regular bed or ICU) resulting from the dynamics induced by the three exposure scenarios.

**Fig 10:**
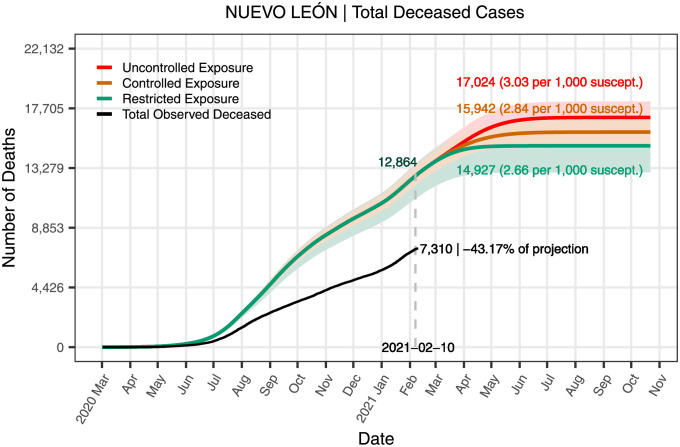
Deceased cases in the state of Nuevo León for the three scenarios. The figure shows the curves of cumulative deaths resulting from the dynamics induced by the three exposure scenarios.

The calculator was run up to 2021–10-16 to compare the results obtained for each scenario. At this date, the dynamics generated for each of the three scenarios have arrived at their endpoint. The projected infected, hospitalized, and deceased cases are determined and plotted for each scenario to contrast the three scenarios; these are possibly the most important results for the management of the pandemic. Figs 8–10 depict such information, while Table 8 summarize these and other relevant aspects of the pandemic. It must be mentioned that the first officially infected person in Nuevo León was identified on 2020–03-01; however, the calculator is run from the moment there were at least 20 cases until 2021–10-16. The information used to construct the tables and figures goes from the pandemic’s beginning in Nuevo León until February 10th, 2021, except for the variable mobility. After that day, the three scenarios are used to project the dynamic of the pandemic.

**Table 8:** Nuevo León’s approximated quantities at the pandemic’s endpoint.

NUEVO LEÓN—*Total Population* 5,610,153			
2021–10–16	Restricted Exposure	Controlled Exposure	Uncontrolled Exposure
**Cumulative Infectious Cases**			
*Asymptomatic*	2,054,290	2,251,812	2,460,756
*Mild Symptoms*	1,336,426	1,464,909	1,600,818
*Hospitalized (Regular Bed)*	27,007	29,613	32,396
*ICU*	7,082	7,511	7,968
*0–9 years*	558,564	612,240	669,024
*10–59 years*	2,490,850	2,730,152	2,983,311
*60+ years*	375,391	411,453	449,603
*Cumulative cases*	3,424,805	3,753,845	4,101,938
**Cumulative Hospitalized Cases**			
*Hospitalized (Regular Bed)*	27,007	29,613	32,396
*ICU*	7,082	7,511	7,968
*0–9 years*	529	579	633
*10–59 years*	16,999	18,352	19,796
*60+ years*	16,561	18,193	19,935
**Cumulative Deceased Cases**			
*0–9 years*	40	44	48
*10–59 years*	4,502	4,754	5,022
*60+ years*	10,385	11,145	11,955
*Total deaths*	14,927	15,943	17,025
**Susceptible and Recovered Population**			
*Susceptibles*	2,185,348	1,856,307	1,508,214
*Recovered*	3,409,878	3,737,902	4,084,913

Fig 8 shows the active cases for the three scenarios, including its 95% confidence bands. The active cases are the number of cases that continue being infectious; that is, it results from the total number of confirmed cases minus the total number of recovered cases and deaths. The figure also includes the number of the active cases at the peak of the pandemic at the date the data is presented; for this case, the scenarios have the same maximums.

Fig 9 shows the daily active hospitalizations resulting from the dynamics induced by the three exposure scenarios with their corresponding 95% confidence bands. For comparison, the figure also includes the reported active hospitalizations on the last day of this study. It also indicates the number of the projected active hospitalizations on the peaks of each scenario. As before, the three scenarios display the same maximum.

Fig 10 presents the curves of cumulative deaths resulting from the dynamics induced by the three exposure scenarios and their 95% confidence bands. For comparison, the figure also shows the confirmed reported deaths in black and the difference, expressed as a percentage, between the projected and reported deaths on the last day of this study. The figure also includes the total projected deaths at the end of the pandemic. The subreport of COVID-19 related deaths [41, 42] explains the difference between the number of confirmed reported deaths and the deaths projected by the model.

The previous figures show that the same peaks for the active infected and hospitalized cases. Nevertheless, as show in Table 8, there is a significant difference in many important variables between the scenarios.

**Fig 11:**
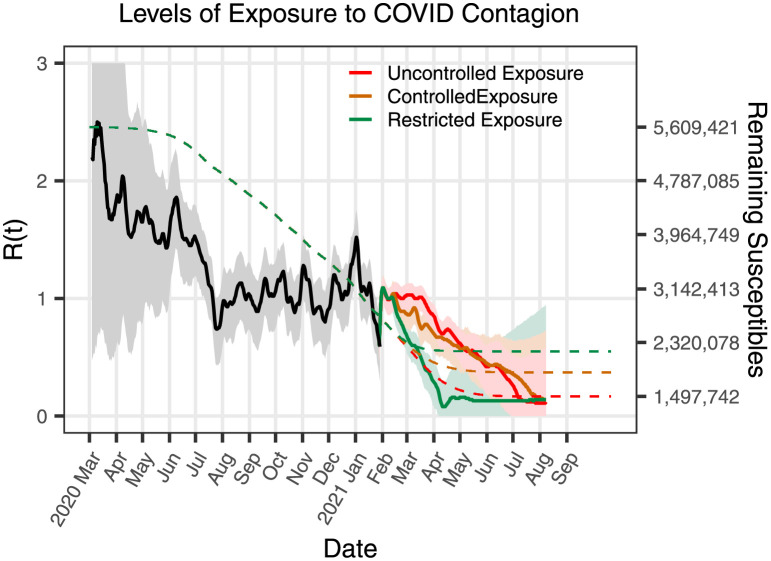
*R*_*t*_, scenarios, and susceptible population for the state of Nuevo León. The figure shows the comparison of the curves of *R*_*t*_ produced by the three scenarios plotted.

Fig 11 illustrates the results obtained using the proposed methodology for the calculation of the effective reproduction number *R*_*t*_ with the information available. Recall that *R*_*t*_ is computed using [15] in the infected cases generated by the EC under the three possible scenarios. The drop in value observed in the *R*_*t*_ curves reflects the fact that the information of emerging conditions has not yet been included. At the time of this study, the required information was not available. This behavior can be corrected, for instance, by just changing the weighting between the scenario and the susceptible population in the projection of *R*_*t*_. However, as the missing data becomes available and is updated, it is simpler to incorporate into the model the new dynamics corresponding to reinfections, vaccination, and new variants.

Finally, Table 8 contains the projected infectious, hospitalized, and deceased cases at the pandemic’s endpoint for the three scenarios. The information is presented at different levels of disaggregation. The table also contains the total susceptible and recovered population.

### National

This section presents the national results generated by the EC for the entire country of México. These findings are obtained by generating, for each of the 32 Mexican states, exposure scenarios analogous to those presented in Fig 6. Since we consider them the most plausible scenarios for México’s at this moment, only the controlled and uncontrolled exposure scenarios are produced. Those scenarios are used to project curves of *R*_*t*_ following the procedure previously described. Thus, for each state are obtained curves for the evolution of infected, hospitalized, and deceased cases. A picture of the pandemic’s evolution at the national level is obtained by aggregating the information of the 32 states.

The significant heterogeneity between the states makes us generate the national information in the manner described above. This diversity not only reflects differences in economic, social, and political aspects; it also influences the public policies implemented to limit the pandemic in each state. Thus, it seems inappropriate to generate national scenarios as it was done in the state of Nuevo León’s case. The national results appear summarized in Table 9. They are analogous to those determined and commented for Nuevo León.

**Table 9:** México’s approximated quantities at the pandemic’s endpoint.

NATIONAL— *Total Population* 127,792,286		
2021–11–26	Controlled Exposure	Uncontrolled Exposure
**Cumulative Infectious Cases**		
*Asymptomatic*	44,107,455	58,397,234
*Mild Symptoms*	28,626,230	37,859,055
*Hospitalized (Regular Bed)*	812,863	1,159,729
*ICU*	235,355	342,319
*0–9 years*	12,529,090	16,754,554
*10–59 years*	52,926,729	69,955,889
*60+ years*	8,326,084	11,047,894
*Cumulative cases*	73,781,903	97,758,337
**Cumulative Hospitalized Cases**		
*Hospitalized (Regular Bed)*	812,863	1,159,729
*ICU*	235,355	342,319
*0–9 years*	57,832	61,052
*10–59 years*	451,290	640,172
*60+ years*	539,096	800,823
**Cumulative Deaths**		
*0–9 years*	4,425	4,794
*10–59 years*	194,422	277,398
*60+ years*	330,270	474,068
*Total deaths*	529,118	756,261
**Susceptible and Recovered Population**		
*Susceptibles*	54,076,410	30,096,612
*Recovered*	73,186,758	96,939,414

## Conclusion

The present work introduces an EC to describe and project the evolution of a pandemic/epidemic. The parametrization it employs is mostly based on the public information available for México at the time the EC was used. This EC constitutes a tool for the regional management of the phenomena. Even though the fact that, in México, authorities have only provided information of infected cases at the hospital- regular beds and ICU- and ambulatory patients, the EC allows approximating the total number of infected cases, including the asymptomatic and people with mild symptoms at home. The EC also allows disaggregating the information by age group and severity of the infection in the different regions and permits to get a global (national) picture from crosses of the EC-generated information.

The EC permits to project the evolution of the pandemic/epidemic from exposure scenarios. The procedure is based in the projection into the future of the effective reproduction number *R*_*t*_ under those exposure scenarios. These scenarios intend to reflect those aspects that impact the pandemic dynamics, such as the generalized use of face masks, confinement policies, and social distancing. The scenarios may also include the effects of vaccination or the presence of new strains of the virus.

The outputs of the EC can assist policymakers in the decision-making process in regions where the information regarding an outbreak is limited. Since the EC can provide regional approximations and projections of quantities like the total infected, hospitalized, and deceased cases, it can assist in identifying high-risk zones and the best ways to allocate public resources to attend the pandemic. For instance, it can help determine if the public hospitals require more beds in ICU for infected patients. It also may help evaluate public policies to treat the outbreaks.

It is worth mentioning that the number of COVID-19 deaths approximated by the national EC coincides with the most recent estimates of the excess mortality for México, which include data that was not available when this work was submitted. On the other hand, the total projected COVID-19 deaths are in line with estimates by independent sources, even though we do not consider new events like new variants, vaccination, and reinfection. For instance, the average number of deaths between both scenarios is approximately 640,000, while the University of Washington [43] predicts between 710,000 and 640,000 deaths. The difference might come from the way different scenarios are defined.

As any model, the EC can be improved. As future work, it remains to include the effect of the most recent variants that have appeared- as the delta variant-, the vaccination public policies, and possible reinfections. These aspects would require a new parametrization of the EC, including new weights in the projection of *R*_*t*_.

## Supporting information

S1 Appendix Supplemental material on a multi-source global-local model for epidemic management. The appendix contains a note on the latency and incubation periods and an exercise to predict *R*_*t*_ using mobility through a regression model with ARMA errors. It also presents the application of the EC proposed in this work to the México City Metropolitan Area, officially known as Valle de México; this last exercise intends to show that the EC also works at different levels of geographic disaggregation.
(PDF)
