# Supplementary material for: A multi-source global-local model for epidemic management
Source: PLoS One. 2022 Jan 12;17(1):e0261650. doi: 10.1371/journal.pone.0261650 (PMC8754321; doi:10.1371/journal.pone.0261650)
Supplement: S1 Appendix — The appendix contains a note on the latency and incubation periods and an exercise to predict Rt using mobility through a regression model with ARMA errors. It also presents the application of the EC proposed in this work to the México City Metropolitan Area, officially known as Valle de México; this last exercise intends to show that the EC also works at different levels of geographic disaggregation. (PDF) [file pone.0261650.s001.pdf]

S1 Appendix: Supplemental material on *A Multi-Source Global-Local model for epidemic management*

J. Ulises Márquez Urbina[1,2], L. Leticia Ramírez Ramírez[3*], Graciela González Farías[3], D. Iván Rodríguez González[4],

1 Unidad Monterrey, CIMAT, Monterrey, N.L. México.
2 Consejo Nacional de Ciencia y Tecnología, México City, CDMX, México.
3 Dep. Probability and Statistics, CIMAT Guanajuato, Guanajuato, Gto. México.
4 Department of Technological Services, CIMAT, Guanajuato, Gto, México,

* leticia.ramirez@cimat.mx

A note on the latency and incubation periods

To introduce the time lag between the latent and incubation endings [1–3] and the time that takes a person to seek for medical help and being hospitalized (status I_{ij} for $j = 3, 4$), we consider that $I^\star$ includes all of the individuals with status I' and I, but it should exclude those in that are hospitalized (I_{ij}, $j = 3, 4$). This is done to reflect the fact that those hospitalized are not likely to infect new people. Thus,

$$I^\star = \sum_{i=1}^{3}\sum_{j=1}^{4} I'_{ij} + \sum_{i=1}^{3}(I_{i1} + I_{i2}).$$

That is, we consider that individuals begin to be infectious before presenting symptoms and that those who are asymptomatic or present mild symptoms do not decrease significantly the number of contacts they had before being infected.

Predicting R_t with mobility

In the following, it is illustrated the results of predicting R_t using a regression with ARMA errors, as discussed in the main manuscript. Fig 1 presents a graph of the historical calculation of R_t for the state of Baja California Sur. The confidence bands are calculated as it was proposed in the original reference [4].

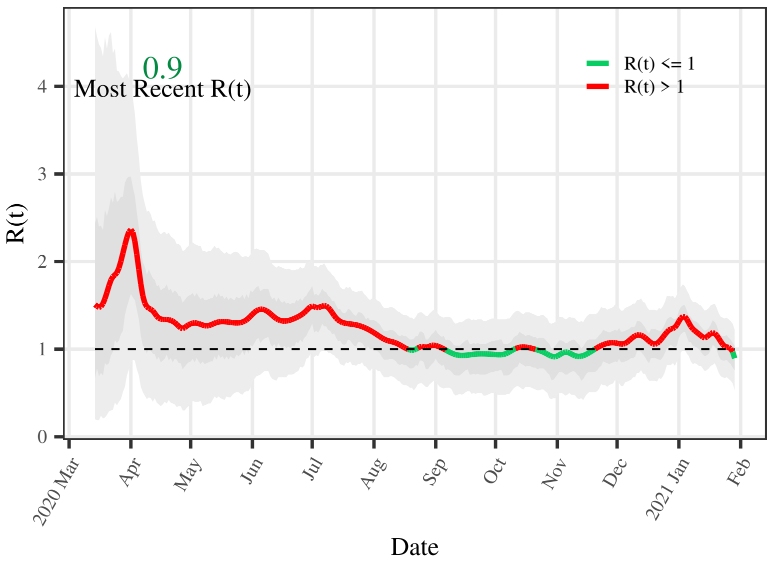

Fig 1. R_t's historic behavior for Baja California Sur.

The effective reproduction number R_t can be predicted from the rate of mobility previously described using a linear regression with Autoregressive Moving Average (ARMA) errors. However, it is well known that these models can provide only short-term predictions. Fig 2 shows an example of such prediction.

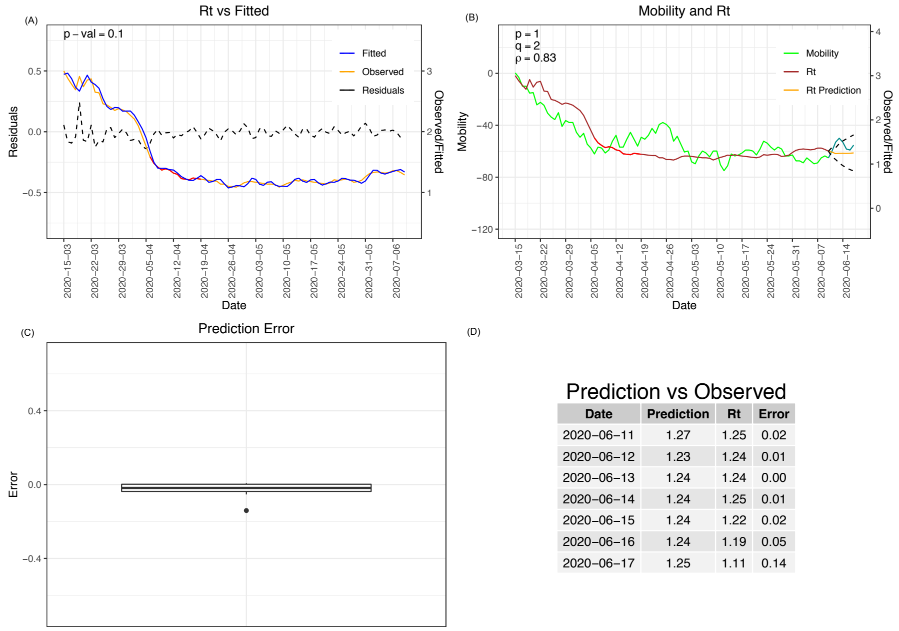

Date	Prediction	Rt	Error
2020−06−11	1.27	1.25	0.02
2020−06−12	1.23	1.24	0.01
2020−06−13	1.24	1.24	0.00
2020−06−14	1.24	1.25	0.01
2020−06−15	1.24	1.22	0.02
2020−06−16	1.24	1.19	0.05
2020−06−17	1.25	1.11	0.14

Fig 2. R_t prediction vs. mobility. The figures and the table show the adjustment and prediction at seven days for R_t using the mobility and an ARMA error models. The top figures show in red R_t and the mobility for the periods of Holy Week and Easter.

As it has been noted by authors such as [3,5,6], it was to be expected that the effective reproduction number R_t would be related to mobility. Such dependence can be

observed in Fig 2: the graph on the upper left (A) shows the estimated adjustment of R_t obtained with a regression model with ARMA errors; in it, the blue line and the yellow line follow one another. On the other hand, it shows the residuals of the adjustment and it can be observed that outside of the initial points it behaves like white noise. In addition, and only for visualization, the graph on the upper right (B) of Fig 2 shows the mobility and the adjustment for the regression of R_t. The upper right figure (B) also presents the prediction of one week generated by the regression; for this, a week of data is saved to see the prediction's quality. This graph also shows the correlation between mobility and R_t calculated in this period, $\rho = 0.83$. The absolute prediction error is shown in the lower table (D) and at the histogram in the lower graph (C) of Fig 2. The behavior observed in Fig 2 is consistent in almost all of the strata that were studied. Some of the special cases that did not follow well these correlations between R_t and mobility were initially caused by the fact that they belonged to places with few contagions or places in which effective distancing measures were not imposed in their locations.

Valle de México's results

The projections of the curve R_t for México City Metropolitan Area, officially known as Valle de México, are presented in the following. As in the case of the state of Nuevo León, these projections are calculated from three scenarios, which appear in Fig 3. These scenarios are analogous to those used in the case of Nuevo León: a situation where strict restrictive measures are imposed to control the pandemic (restricted exposure); a scenario where generalized measures of control are applied (controlled exposure); and a situation where no generalized measures of control are applied (uncontrolled exposure). As in the case of Nuevo León, the information about the local evolution of the pandemic is determined using the curves of R_t in conjunction with the epidemiological calculator.

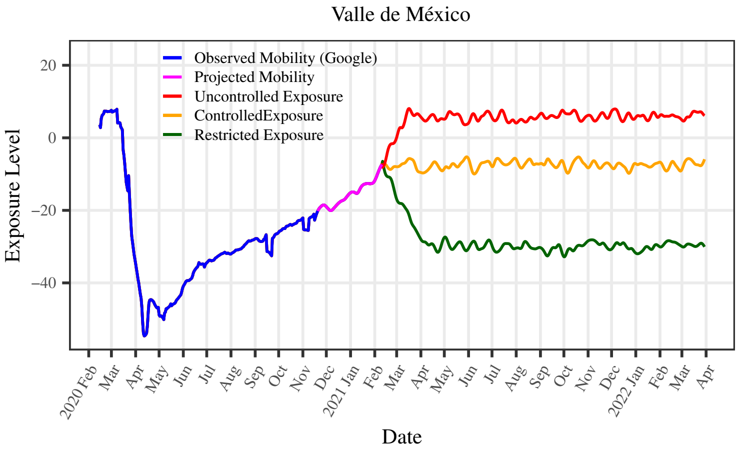

Fig 3. Exposure scenarios for Valle de México. The figure presents the observed mobility up to November 19th, 2020; from that point, it shows a projection of the mobility until the most recent date of this study (February 10th, 2021), followed by the three exposure scenarios.

Under the scenarios illustrated in Fig 3, the following presents some of the illustrative results produced by EC for Valle de México's metropolitan area. To compare the results obtained for each scenario, the calculator is run from the moment there are at least 20 cases in Valle de México until 2021-10-16. At this date, the dynamics

generated for each of the three scenarios have arrived at their endpoint. The information used to construct these tables and figures goes from the beginning of the pandemic in Valle de México until February 10th, 2021, except for the mobility. As the mobility index, it is used the observed mobility in conjunction with its projection (see Fig 3). It is important to remember that EC only considers and employs the information up to February 10th, 2021. From this date on, many significant changes will be going on, like new variants, vaccination programs, and reinfections. The EC can take this information. However, there was no public information on these aspects for Mexico when this work was subject to the first revision.

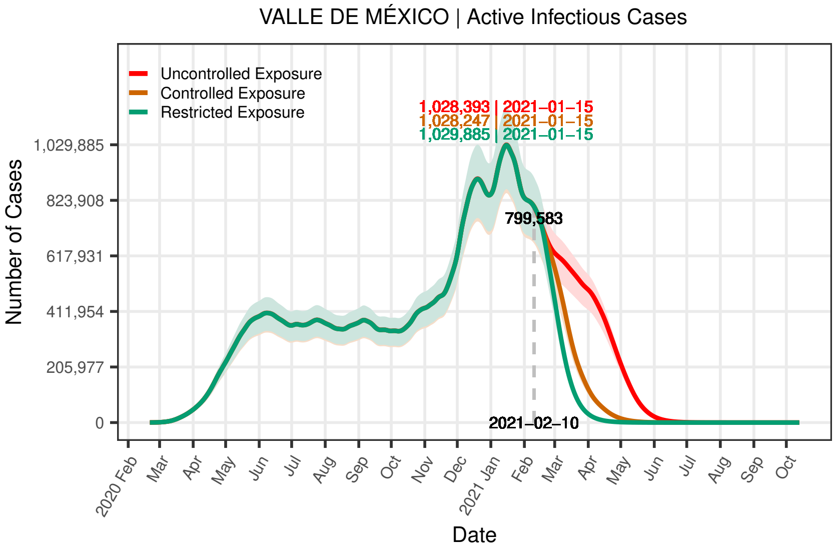

Fig 4. Active cases in Valle de México for the three scenarios. The figure shows the curves of daily active infected cases resulting from the dynamics induced by each exposure scenarios.

Fig 4 shows the active cases for the three scenarios, including its 95% confidence bands. The figure also includes the number of active cases at the pandemic's peak of each scenario; for this case, the scenarios have close maximums.

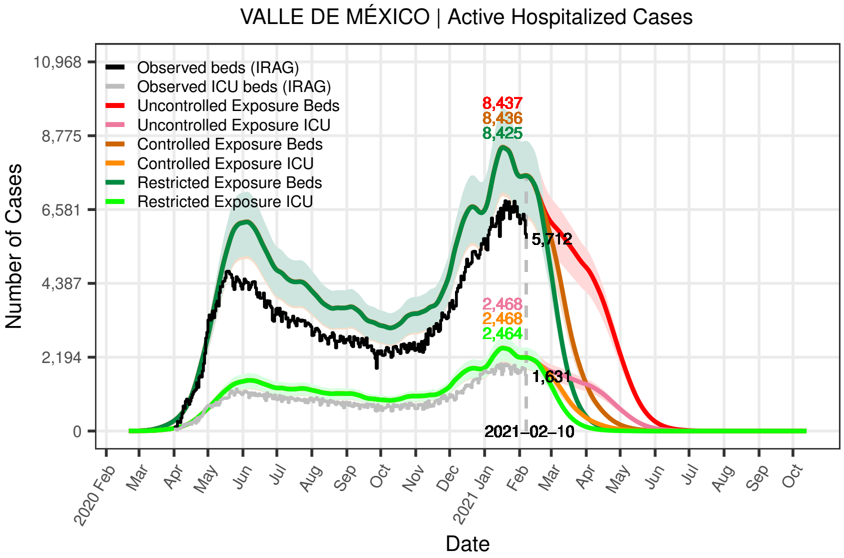

Fig 5. Active Hospitalized cases in Valle de Mexico for the three scenarios. The figure shows curves for the active hospitalized cases for each type of hospitalization (regular bed or ICU) resulting from the dynamics induced by the three exposure scenarios.

Fig 5 shows the daily active hospitalizations resulting from the dynamics induced by the three exposure scenarios with their corresponding 95% confidence bands. For comparison, the figure also includes the reported active hospitalizations on the last day of this study. It also indicates the number of the projected active hospitalizations on the peaks of each scenario. As before, the three scenarios display small differences at the maximums.

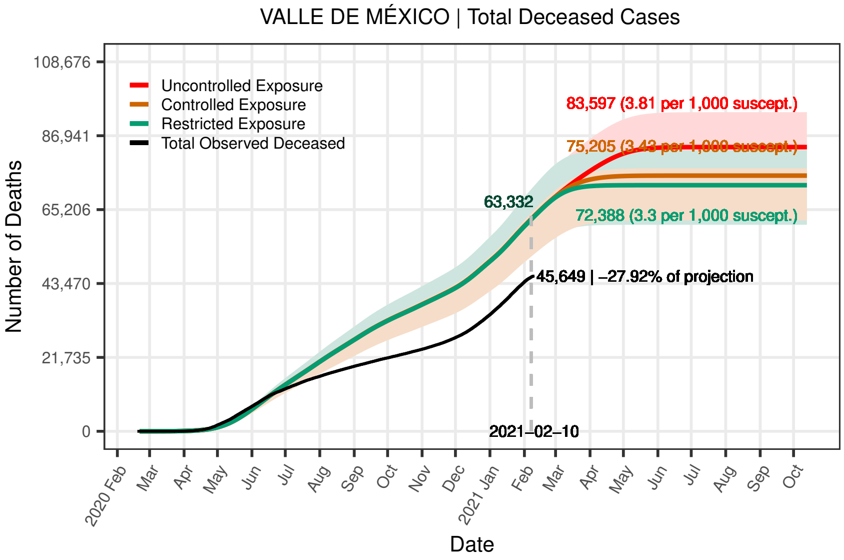

Fig 6. Deceased cases in Valle de México for the three scenarios. The figure shows the curves of cumulative deaths resulting from the dynamics induced by the three exposure scenarios.

Fig 6 presents the curves of cumulative deaths resulting from the dynamics induced by the three exposure scenarios and 95% confidence bands. For comparison, the figure also shows the confirmed reported deaths in black and the difference, expressed as a percentage, between the projected and reported deaths on the last day of this study. The figure also includes the total projected deaths at the end of the pandemic. As explained in the main manuscript, the subreport of COVID-19 related deaths explains the difference between the number of confirmed reported deaths and the deaths projected by the model.

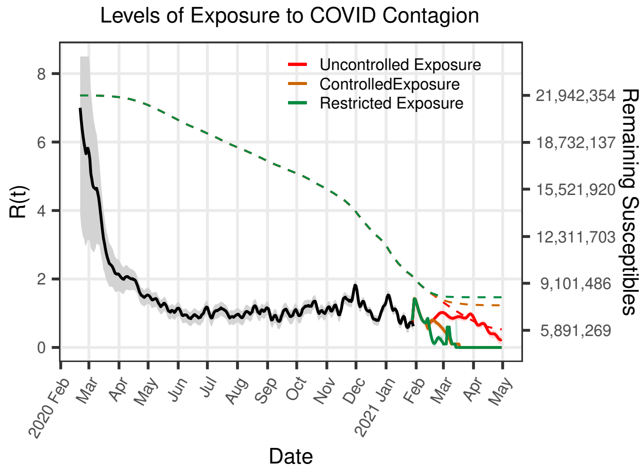

Fig 7. R_t, Scenarios and Susceptible Population for Valle de México. The figure shows the comparison of the curves of R_t produced by the three scenarios plotted.

Fig 7 illustrates the results obtained using the proposed methodology for calculating the effective reproduction number R_t with the available information. The drop in value observed in the R_t curves reflects that the information of emerging conditions is not included. At the time of this study, the required information was not available. As the missing data on reinfections, vaccination, and new variants become available, this behavior can be corrected, as explained in the main manuscript.

Finally, Tab 1 contains the projected infectious, hospitalized, and deceased cases at the pandemic's endpoint for the three scenarios. The information is presented at different levels of disaggregation. The table also contains the total susceptible and recovered population.

Table 1. Valle de México's estimated quantities at the pandemic's endpoint.

**VALLE DE MÉXICO** — *Total Population* **21,942,666**			
**2021-10-13**	**Restricted Exposure**	**Controlled Exposure**	**Uncontrolled Exposure**
**Cumulative Infectious Cases**			
Asymptomatic	8,222,388	8,549,740	9,578,655
Mild Symptoms	5,451,384	5,668,391	6,350,445
Hospitalized (Regular Bed)	114,363	118,873	133,332
ICU	31,934	33,223	37,353
0-9 years	1,915,559	1,991,816	2,231,551
10-59 years	10,103,543	10,505,752	11,770,193
60+ years	1,800,967	1,872,659	2,098,042
Cumulative cases	13,820,069	14,370,227	16,099,785
**Cumulative Hospitalized Cases**			
Hospitalized (Regular Bed)	114,363	118,873	133,332
ICU	31,934	33,223	37,353
0-9 years	1,176	1,223	1,372
10-59 years	77,061	79,963	89,270
60+ years	68,060	70,910	80,043
**Cumulative Deceased Cases**			
0-9 years	120	125	140
10-59 years	28,283	29,373	32,856
60+ years	44,080	45,712	50,941
Total deaths	72,483	75,210	83,937
**Susceptible and Recovered Population**			
Susceptibles	8,122,596	7,572,438	5,842,880
Recovered	13,747,586	14,295,017	16,015,848

References

1. Liu Z, Magal P, Seydi O, Webb G. A COVID-19 epidemic model with latency period. Infectious Disease Modelling. 2020;5:323–337.

2. He X, Lau EH, Wu P, Deng X, Wang J, Hao X, et al. Temporal dynamics in viral shedding and transmissibility of COVID-19. Nature medicine. 2020;26(5):672–675.

3. Linka K, Peirlinck M, Kuhl E. The reproduction number of COVID-19 and its correlation with public health interventions. Comput Mech. 2020;66:1035—1050.

4. Systrom K. Estimating COVID-19's R_t in Real-Time; 2020. Available from: `https://github.com/k-sys/covid-19/blob/master/Realtime%20R0.ipynb`.

5. Badr HS, Du H, Marshall M, Dong E, Squire MM, Gardner LM. Association between mobility patterns and COVID-19 transmission in the USA: a mathematical modelling study. The Lancet Infectious Diseases. 2020;20(11):1247–1254.

6. Miller AC, Foti NJ, Lewnard JA, Jewell NP, Guestrin C, Fox EB. Mobility trends provide a leading indicator of changes in SARS-CoV-2 transmission. medRxiv. 2020;.
